# Can universal access and competition in long-term care insurance be combined?

**DOI:** 10.1007/s10754-015-9163-3

**Published:** 2015-01-30

**Authors:** Pieter Bakx, Frederik Schut, Eddy van Doorslaer

**Affiliations:** 1 Institute of Health Policy and Management, Erasmus University, P.O. Box 1738, 3000 DR Rotterdam, The Netherlands; 2 Department of Applied Economics, Erasmus School of Economics, Erasmus University, P.O. Box 1738, 3000 DR Rotterdam, The Netherlands; 3 Tinbergen Institute, P.O. Box 1738, 3000 DR Rotterdam, The Netherlands

**Keywords:** Risk adjustment, Long-term care, Managed competition, Public insurance, H51, I11, I13, I18, L13

## Abstract

In countries with a public long-term care (LTC) insurance scheme administered by multiple non-competing insurers, these insurers typically lack incentives for purchasing cost-effective LTC because they are not at risk for LTC expenses. Plans to introduce these incentives by allowing competition among risk bearing LTC insurers are likely to jeopardize universal access. Combining universal access and competition among risk bearing LTC-insurers requires an adequate system of risk adjustment. While risk adjustment is now widely adopted in health insurance, LTC-specific features cause uncertainty about the feasibility of risk adjustment for LTC insurance. We examine the feasibility of appropriate risk adjustment in LTC insurance by using a rich set of linked nationwide Dutch administrative data. As expected, prior LTC use and demographic information are found to explain much of the variation in individual LTC expenses. However, we find that prior health care expenditures are also important in reducing predicted losses for subgroups of health care users. Nevertheless, incentives for risk selection against some easily identifiable subgroups persist. Moreover, using prior utilization and expenditure as risk adjusters reduces incentives for efficiency, creating a trade-off between equity and efficiency. To ease this trade-off, data on individuals’ underlying needs for LTC are required.

## Introduction

Worldwide, health policy makers are confronted with ageing populations and rising demand for long-term care^1^ (LTC) and are looking for ways to guarantee access to LTC services in a sustainable way. Barr (2010) argues that there is a strong case for public provision of LTC insurance. Indeed, virtually all OECD countries have at least some publicly provided mandatory coverage against LTC expenditures. Several of these countries have integrated some “medical” LTC services in their public health insurance schemes, e.g. Belgium, Switzerland and the US Medicare and Medicaid programs. Other countries have a separate public LTC insurance scheme, e.g. the Netherlands (since 1968), Germany (since 1995), Japan (since 2000) and South-Korea (since 2002).

Typically, public LTC insurance is provided by public non-competing insurers who are not at risk for the LTC expenses of their enrollees (Costa-Font and Courbage 2012). For instance, in the Netherlands LTC insurance is administered by about 30 regional insurers that are fully reimbursed for the LTC expenses of their clients that are covered by the public scheme. As a consequence, these public insurers have no incentives to secure that high-quality LTC services are provided at low cost. To control expenditures in public LTC insurance, governments have traditionally relied on demand rationing (e.g. means testing, copayments and coverage restrictions), and supply rationing (e.g. price regulation, provider budgets, and capacity restrictions) (Costa-Font and Courbage 2012). Both types of rationing, however, have important drawbacks, which are likely to be exacerbated by the expected increase in demand for LTC. Demand-side rationing may result in access problems for low-income individuals who need LTC; supply-side rationing may result in waiting lists and substandard quality of care.

In several countries, another way to encourage efficient use of LTC has been introduced or proposed: to provide LTC insurers with incentives to contract efficient LTC providers. This could be achieved by putting LTC insurers at risk for providing LTC coverage and allowing them to compete for customers. However, competition among risk-bearing insurers is likely to jeopardize universal access because LTC expenses are typically high and correlated over time (Van Barneveld et al. 1997) and consequently actuarially fair premiums will be unaffordable to many people needing LTC.

To combine competition with universal access in social health insurance markets, several countries have introduced a system of managed competition in which insurers cannot reject applicants and are required to charge community-rated premiums to all applicants. To guarantee affordable access to coverage, insurers receive risk-adjusted premium subsidies^2^ that eliminate or at least reduce incentives to increase profits through risk selection^3^. These subsidies reduce differences in expected costs between individuals and thus make all applicants equally attractive for an insurer (Enthoven 1988; Van de Ven and Ellis 2000).

Managed competition has been proposed and implemented to ensure equitable access to public LTC insurance (Medicaid) in the US and increase its efficiency (Lucas 1996). In Switzerland and Belgium, medical LTC is integrated in social health insurance coverage that is offered by risk-bearing competing health insurers, although the financial risk for Belgian insurers is quite limited (OECD 2011; Schokkaert and van de Voorde 2011; Weaver 2012; Willeme et al. 2012). Recently, the Dutch government launched a similar proposal. According to this proposal, managed competition will be introduced for home care in 2015 and after 2 years insurers should bear the full risk for covering home health benefits. To this end, coverage for home care is included in the social health insurance benefit package. Managed competition may also be introduced for nursing home care after 2017 (Rijksoverheid 2013).

An appropriate system of risk-adjusted premium subsidies is crucial to safeguarding universal access in a competitive LTC insurance market. However, whereas risk adjustment in health insurance has been studied extensively, empirical research on risk adjustment in LTC insurance is nearly nonexistent. The aim of this paper is to examine how and to what extent a system of risk adjusted subsidies can reduce the financial incentives for risk selection in LTC insurance within the context of managed competition. To this end, the following five questions are addressed: (1) How do LTC expenditures differ from expenditures on medical care and how do these differences affect the options to use risk adjustment to reduce risk selection? (2) What are the predicted losses and gains on LTC for insurers in case of annual contracts, community rating and no risk adjustment? (3) To what extent are the predicted losses and gains reduced by the most comprehensive risk-adjustment model based on data on: (i) demographic characteristics, (ii) prior LTC use and (iii) prior health care expenditures (HCE) and inpatient hospital diagnoses? (4) What is the contribution of each of these sets of risk adjusters to the reduction of the predicted losses and gains in the most comprehensive risk-adjustment model? (5) How are the predicted losses and gains affected when the risk adjusters that provide substantial perverse incentives to insurers are removed from the risk adjustment model?

## What is already known about risk adjustment in long-term care?

The experience with risk adjustment in health insurance cannot be readily used to develop an appropriate risk adjustment system for LTC insurance. LTC expenditures differ from health care expenditures (HCE) in at least two important aspects (Van de Ven 2005). First, LTC expenditures are concentrated among a limited group of beneficiaries, and are, conditional upon use, high and stable over time. Consequently, in the absence of risk adjustment, risk selection based on prior expenditures is much easier in LTC insurance than in health insurance. Second, the availability of informal care is expected to have a much larger impact on LTC expenditures than on HCE. But there is little experience with including informal care availability in risk adjustment and the availability of informal care is difficult to quantify with administrative data. Hence, differences in informal care availability cannot be fully captured by the risk adjustment formula.

Little is known about how these issues can be dealt with and about how to design appropriate risk adjustment for LTC insurance. To date, there is only one study about the feasibility of risk adjustment in Dutch LTC insurance (Van Barneveld et al. 1997). With prior LTC expenditure as a risk adjuster and using data from one sickness fund, Van Barneveld et al. (1997) examine the remaining potential for risk selection in the Dutch public LTC insurance scheme. They find an $\hbox {R}^{2}$-statistic of 0.90, which indicates that LTC expenditures are highly predictable at the individual level when information on prior expenditures is available.

Using prior expenditures as a risk adjuster means that the insurer will be partly or fully compensated for higher expenditures through higher future risk-adjusted capitation payments. This compensation may give insurers incentives for overprovision. Hence, compared to the situation of capitation payments that are not based on prior expenditures, insurers face fewer incentives for an efficient provision and allocation of LTC. Marchand et al. (2003) show that despite this drawback, if insurers compete on quality, they receive stronger incentives to be efficient when risk adjustment is based on prior expenditures or prior use than when they are fully reimbursed for all expenditures.

Several studies on risk adjustment in US Medicare and Medicaid have tackled similar issues. While the Medicare benefit package does not include LTC, the target population of Medicare is similar and studies on risk adjustment in Medicare therefore provide a number of relevant insights. First, risk adjustment for Medicare Advantage plans and for the Medicaid Program of All-Inclusive Care for the Elderly (PACE) takes into account frailty as measured by the number of activities of daily living (ADL) problems; a risk adjustment model without frailty was found to systematically underestimate expenditures for the frail elderly and might therefore induce risk selection against this group (Kautter et al. 2009). Second, the relationship between health care use in the past, demographic characteristics and future health expenditures changes upon institutionalization and it is different for those who became eligible for Medicare by reaching the age of 65 and those who became eligible because they were disabled (Pope et al. 2004). This finding implies that risk adjusters should be interacted with institutionalization and age. Third, incentives for risk selection persist despite extensive risk adjustment: while risk selection on expected costs decreased after expanding the risk adjustment formula beyond age and gender, insurers now select profitable enrollees by focusing on characteristics not included in the model. Consequently, the Medicare program has become more expensive spending on those in good health increases vis-à-vis spending on those in bad health (Brown et al. 2011).

Outside the US, experience with risk adjustment in LTC insurance is limited to Switzerland and Belgium. In these countries, medical LTC is included in social health insurance. In Switzerland, the risk adjustment formula comprises age, gender and a dummy variable accounting for a recent stay of at least three days in a hospital or a LTC facility (Von Wyl 2014). This dummy variable is likely to pick up some of the variation in expected LTC expenditures. The Belgian risk adjustment formula includes more LTC-specific risk adjusters. The capitation payment is adjusted for receipt of certain allowances (e.g. for handicapped or because of a need for assistance) or nursing care at home during 3 months (category B or C on the Katz-scale (Katz and Akpom 1976)). In addition, the risk adjustment formula includes a number of indicators related to LTC use, e.g. living alone, being widow/widower, physiotherapy for a severe illness, and Parkinson’s disease (Schokkaert and van de Voorde 2011). While the Belgian risk adjustment formula is more sophisticated than the one used in Switzerland, the financial risk is much more limited for Belgian than for Swiss health insurers (Schokkaert and van de Voorde 2011; Paolucci et al. 2007). Therefore, risk selection against LTC patients appears to be financially attractive in Switzerland but not in Belgium. It is, however, unclear whether the more sophisticated Belgian model would suffice to prevent risk selection if financial risk for insurers were expanded.

## Data and methods

### Data

We use information from five nationwide administrative registries and one survey which are all linked by Statistics Netherlands at the individual level^4^ . The administrative data would be readily available if risk adjustment were implemented and include (1) health care expenditures in 2000–2004 from the health insurance data collected by Vektis; (2) use of LTC in 2004 and 2005, which includes home care, social assistance, assistance with activities of daily living and inpatient stays in either a residential home or a nursing home and which comes from the Central Administration Office of the LTC insurance scheme (CAK); (3) hospital admissions in 2002, 2003 and 2004 from the hospital discharge register (LMR); (4) demographic information for 2004 from the municipal register (GBA) and (5) mortality from the cause-of-death registry (CBS). In addition, the General Survey of Living Conditions (POLS) held in 2004 provides details on health, disability, and other individual characteristics for a randomly drawn, representative sample of the non-institutionalized population. Prior health care expenditures are registered for sickness fund enrollees only (two-thirds of the population)^5^ and LTC use is registered for adults only ($\ge$18 years of age); the other administrative data sets comprise the entire Dutch population.

The sample was further reduced for two reasons. First, the records for one third of those eligible for sickness fund coverage cannot not be linked. Second, 1.7 % of the sample was excluded because of item non-response which always was the result of missing co-residence status. As a result, the final sample consists of individuals who were insured through a sickness fund, did not die in 2004 and whose records could be linked to the municipality register. The total study population was 5,719,934, which is 45 % of the Dutch adult population in 2004. From this subset of the population, 7,790 individuals were included in the 2004 POLS survey; 3,619 of these respondents also completed the more specific health module.

### Methods

A good risk adjustment system should reduce insurers’ incentives for risk selection while maintaining their incentives for efficiency. Ideally, after risk adjustment there are no easily identifiable subgroups for which insurers are undercompensated or overcompensated. In addition to an accurate prediction of individual expenditures, good risk adjusters should provide appropriate incentives and should be administratively feasible (Van de Ven and Ellis 2000). Partly following Beck et al. (2010) and Shen and Ellis (2002) among others, we identify the extent to which a risk adjustment model can reduce incentives for risk selection in three steps. First, we measure the insurers’ incentives to select against subgroups^6^ based on individual characteristics in case of community-rated annual contracts but in the absence of risk adjustment. To quantify the insurers’ incentives for risk selection, we calculate the difference between the average actual expenditures by subgroup and the average expenditures for the entire population in 2005. We consider the incentives for risk selection to be strong when the number of users in the subgroup is substantial $(>$300), the predicted loss for a person in this group—the difference between observed expenditures for this subgroup and average expenditures for the entire population—is large ($>$1,000 euro) and significantly $(p < 0.05)$ different from zero. When these criteria are met, the benefits of risk selection are likely to exceed the costs and therefore the subgroup is included in the risk-adjustment model.

Second, we build the full risk adjustment model in a stepwise manner to examine to what extent each set of individual characteristics contributes to explaining individual variation in LTC use. To this end, we estimate a series of four models. We first test the impact of a basic model based on demographic characteristics on the predicted loss for all subgroups. Next, we add subgroups based on (i) prior LTC use, and (ii) prior health care expenditures and hospital admissions to this basic model variables. The full model includes all subgroups that were identified in the first model. For each risk adjustment model, the remaining predicted loss is the difference between the observed expenditures for these subgroups and the expenditures predicted by the risk adjustment model.

Third, for each subgroup that is included in the full model, we assess the impact of including this subgroup in the risk adjustment formula on the insurers’ incentives for efficiency—a commonly used selection criterion (see e.g. Van Kleef and Van Vliet (2010), Van de Ven and Ellis (2000) and Pope et al. (2000)). Subgroups that are likely to have a negative impact on the insurers’ incentives for efficiency are those for which conditions of eligibility can be easily manipulated by insurers and for which it is highly attractive for them to do so. Manipulation may be financially attractive when the expected benefits exceed the costs, which consist of the required effort and the cost of the additional treatment that the enrollee is required to receive to be eligible for the subgroup. Excluding these subgroups from the full model results in an incentive compatible risk adjustment model. This third step thus sheds light on the tradeoff between creating incentives for efficiency and incentives for risk selection.

All five models described above are estimated by ordinary least squares regression (OLS) in order to facilitate interpretation of the results (Van de Ven and Ellis 2000)^7^. Moreover, all current Dutch risk adjustment models use OLS, so using OLS increases the comparability and compatibility with these models.

The POLS sample was very small compared to the population of sickness fund enrollees and therefore the subgroups based on detailed information about health status, disability and socio-economic status from the POLS survey are not included in the risk adjustment model. Instead these subgroups are used as a benchmark to evaluate the impact of the risk adjustment model on incentives for risk selection.

### Variables

In each of the models, the dependent variable measures public LTC expenditures in 2005. In case the individual dies in 2005, expenditures are annualized by dividing expenditures by the share of the year the individual was alive. The data set provides information on the quantity of LTC that was provided in kind, which was 95 % of the publicly financed LTC in the Netherlands in 2006^8^ (CVZ 2011). The quantities provided, i.e. days institutionalized or hours of home care, are multiplied by the maximum prices as set by the government in order to calculate expenditures; co-payments are not taken into account. The data contains information about institutional care use in 2004 and 2005 and about all use of six types of home care in 2004. For 2005, the data contained information about use of only four out of six types of home care^9^.

The set of subgroups that make up the basic model are based on three demographic characteristics: age, gender and co-residence, i.e. whether someone lived in a single-person household. Age and gender are the backbone of any risk-adjustment model, while co-residence proxies informal care availability. Informal care availability is an element of the eligibility assessment procedure for homecare (CIZ 2005) and formal LTC use is known to be correlated with informal LTC use (Bonsang 2009; Van Houtven and Norton 2004).

The subgroups of LTC users are based on prior LTC *use* rather than *expenditures* because using prior LTC use as a risk adjuster rewards insurers for negotiating lower prices with providers. Subgroups are created for each type of home care and each type of institutional care separately. Each of the subgroups of home care users consists of individuals who used this specific type of home care at least 1 hour per week on average. In selecting subgroups of institutional care users, we aim at balancing responsiveness to changes in LTC use against incentives for overreporting and oversupply resulting from the (partial) reimbursement of additional expenditures in the future. Therefore, for each of the four types of institutional care, four subgroups are generated consisting of individuals who stayed in an LTC institution for $\ge$1 day, 91–180 days, 181–365 days, and the entire year (366 days), respectively. These subgroups reflect differences in expected future expenditures between long-term and short-term residents: future expenditures are positively correlated with the number of days that the individual is institutionalized. Furthermore, following Van Barneveld et al. (1997), two subgroups are created consisting of patients who received home care and institutional care, respectively, on the last day of 2004, which shows the size of the predictable loss for enrollees who only use a very small amount of LTC in the prior year.

We also include subgroups based on prior HCE. Each of these subgroups measures health care expenditures^10^ that are associated with LTC use: expenditures on hospital and outpatient care, prescription drugs, paramedical care, transportation, and durable medical equipment. For each of these categories, three subgroups are constructed that consist of persons who are among the 15 % who had the highest expenditures during the last year (omitted for hospital and outpatient expenditures), during each of the last 3 years, and during each of the last 5 years. Because the data only includes HCE covered by sickness funds, we also include a variable indicating which persons were not insured through a sickness fund in one of the 4 years preceding 2004. If someone was no longer registered with a sickness fund during a year, e.g. because of losing his/her eligibility status due to exceeding the income threshold, and hence is not in the data set for the entire year, expenditures are annualized.

In addition to the subgroups based on prior HCE, we also create subgroups based on hospital admissions because information on hospitalization and diagnosis information may help to predict LTC use (Wong et al. 2010). Subgroups are based on 94 diagnoses (based on a grouping algorithm of ICD-9 codes, see Polder et al. 2002) and on 48 types of treatments (based on ICD-9-CM volume 3 codes) using hospital admission data from 2002–2004. In addition, we create 12 Diagnostic Cost Groups (DCGs). DCGs are used for risk adjustment in the Dutch health insurance scheme and consist of clinically homogenous inpatient diagnoses for chronic health problems that have similar future HCE (Van de Ven and Ellis 2000). Using the ICD-code of the main diagnosis and the medical specialty that set this diagnosis, each individual is assigned to either the reference group (DCG 0)—people with no hospital admission or an incidental admission (e.g. fractures)—or the highest DCG they are eligible for (Rijksoverheid 2005; Prinsze and van Vliet 2007)^11^. We include the DCGs but not the separate subgroups based on diagnoses and treatments in the risk adjustment model because the subgroups based on diagnosis and treatments and the DCGs overlap. Furthermore, the impact of the DCGs on the incentives for efficiency is known to be limited in the context of health insurance (Lamers 1998) while including all subgroups separately will increase incentives for oversupply and over-reporting.

As the administrative data do not provide detailed information on personal characteristics, subgroups based on health, disability and socio-economic characteristics could only be created using the smaller set of respondents that completed the POLS survey. Although it is much smaller and persons in nursing homes are not sampled, this survey allows investigating incentives for insurers to use such questionnaires for risk selection purposes. The same subgroups are used as in De Meijer et al. (2011), who study determinants of LTC expenditures among the elderly, and in Stam and van de Ven (2008), who identify subgroups that generate losses for health insurers. Of these subgroups, only those are selected for which the predicted loss deviates significantly from zero in the absence of risk adjustment. Because the average predicted profit without risk adjustment for the POLS sample and the subsample answering the health module are positive, the predictions for these samples are adjusted by subtracting the mean deviation from zero for the relevant sample multiplied by the ratio of the individual’s observed expenditures to the sample mean observed expenditures in order to ensure that the average predicted profit was zero for this subsample.

## Results

### Descriptive statistics

**Fig. 1:**
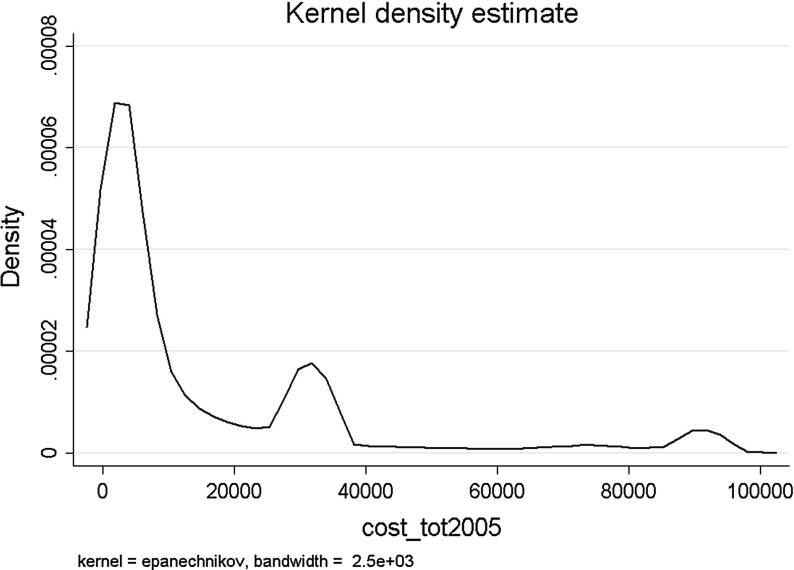
Distribution of LTC expenditures in 2005 of LTC users in 2005

Figure 1 and Table 1 show that the distribution of LTC expenditures is highly skewed. The median is at 4,598 euro; 2 out of 3 LTC users spend less than 10,000 euro. Furthermore, there are two spikes, one at 32,000 euro (a full year of care in a residential home) and one at approximately 91,000 euro (a full year of care in a nursing home). The average cost per LTC user (15,677 euro) is much higher than the average cost per user of medical care (about 2000 euro in 2004). Furthermore, LTC expenditures are strongly correlated with prior use of LTC: average LTC expenditures in 2005 are higher for home care users in 2004 than for non-users and highest for nursing home residents in 2004 (Table 1).

**Table 1:** Descriptive statistics population

	Mean	SD
LTC expenditures in 2005	1,159.18	7,564.05
LTC expenditures in 2005 conditional on any use	15,677.04	23,370.78
LTC expenditures in 2005 if no LTC was received in 2004	93.62	1,657.39
LTC expenditures in 2005 if received home care in 2004	9,673.22	18,333.23
LTC expenditures in 2005 if stayed in a residential care facility in2004	31,767.09	15,902.13
LTC expenditures in 2005 if stayed in a nursing home in 2004	61,451.47	35,674.80

### Analysis

The regression analysis reveals that the included covariates explain a large share of the variation in aggregate expenditures of LTC use in 2005: the $\hbox {R}^{2}$-statistics are generally higher than those obtained in similar studies on medical care and mental health care (see e.g. Van de Ven and Ellis 2000)^12^. Most of the explanatory power derives from the demographic variables and prior LTC use. The model that only includes demographics has an $\hbox {R}^{2}$ of 0.23. Including prior LTC use increases the $\hbox {R}^{2}$-statistic to 0.73, while variables related to prior HCE contribute only marginally to the overall goodness of fit, regardless of whether prior LTC use is included. A Copas test (Copas 1983) did not detect overfitting and therefore we do not need to split the sample in two. Nearly all coefficients are significant in each of the models and show the expected sign. The DCGs sometimes violate the monotonicity requirement: being assigned to a higher DCG with a more severe diagnosis does not in all cases lead to a higher capitation payment. This is undesirable as it generates disincentives for providing more care in cases in which more care might be desirable. The most prominent example is DCG 4, which includes diagnoses related to a cardiovascular accident; myocardial infarct; and angina pectoris among other things, and which has the third largest coefficient. These results highlight that the relationship between prior hospital stays and LTC expenditures is different from the relationship between prior hospital stays and HCE, on the basis of which these DCGs were constructed.

#### No risk adjustment model

In case of annual contracts with community rated premiums but no risk adjustment, the predicted losses would be very large for subgroups based on prior LTC use or based on prior health care expenditures (Table 2)^13^. These predicted losses, together with the large size of most of these subgroups (last column), signal that incentives for risk selection against these subgroups would be huge. Other results (available upon request) show that some diagnoses are indicators of a persistent loss: for four diagnoses that yield a large predicted loss in the next year, the predicted loss is still larger than 1,000 euro 2 years later and 3 years later.

**Table 2:** Predicted losses for selected subgroups

	No risk adjustment	Demographic model	Prior LTC model$^{\mathrm{a}}$	Prior HCE and DCG model$^{\mathrm{a}}$	Full model$^{\mathrm{a}}$	Incentive compatible model$^{\mathrm{a}}$	Subgroup size
Demographic information	No	Yes	Yes	Yes	Yes	Yes	
Prior LTC use	No	No	Yes	No	Yes	Yes	
Prior HCE and diagnosis information	No	No	No	Yes	Yes	Yes	
Subgroups of LTC users in prior year							
Personal care	16,992*	8,293*		7,269*			82,116
Nursing	25,799*	17,183*		15,303*			24,727
Nursing home: combined care 1–90 days	31,009*	23,310*		20,888*		21,005*	10,546
Nursing home: combined care 91–180 days	51,350*	42,482*		39,803*			3,853
Nursing home: combined care 180–365 days	71,408*	61,349*		59,290*			4,359
Nursing home: combined care 366 days	85,868*	74,655*		74,693*			12,439
Receiving home care on last day of 2004	10,078*	2,459*		1,669*		876*	172,291
Subgroups based on HCE in 2000–2004							
In top 15 % in prior 3 years: hospital + outpatient care	3,410*	1,804*	278*				137,618
In top 15 % in prior 5 years: hospital + outpatient care	4,031*	2,321*	393*				57,597
Expenditures on transportation in prior year	7,350*	4,271*	582*			916*	201,609
Expenditures on transportation in prior 3 years	9,040*	5,753*	353*				43,615
Expenditures on transportation in prior 5 years	8,565*	5,347*	374*				22,462
DCG 4, e.g. Cardiovascular accident, stroke, angina pectoris	10,172*	6,865*	1,145*				11,358
Subgroups based on diagnosis information from 2004 hospital admission data							
Dementia	30,423*	20,777*	9,255*	20,114*	9,274*	11,692*	821
Hip fracture	21,225*	12,029*	$-$950*	9,167*	$-$1,229*	3,205*	6,433
Chronic ulcers of skin including decubitus	13,421*	8,676*	$-$224	4,514*	$-$1,897*	$-$1,528*	873
Stroke	10,840*	7,288*	1,109*	2,219*	327*	1,271*	11,998
Heart failure	10,054*	3,806*	858*	745*	182	637*	8,147
Diabetes mellitus including diabetic complications	6,478*	4,633*	679*	2,770*	133	231	4,746
Asthma and COPD	6,128*	3,721*	876*	122	$-$78	26	8,035
Subgroups from POLS health survey (n = 4,619)							
Has difficulty to/cannot perform $\ge$1 ADL	16,221*	8,918*	357	7,887*	289	532	90
Cannot perform $\ge$1 ADL	23,365*	13,476*	1,479	11,906*	1,409	1,365	32

$^{\mathrm{a}}$ Cells are empty if variable is included in this extension of the risk adjustment model

*Significant at the *p* $<$ 0.05 level

#### Demographic model

The results for the Demographic Model, which adjusts subsidies for the age, gender and co-residence status of the enrollee, show that including demographic characteristics in the risk adjustment model does not sufficiently reduce the predicted losses for subgroups based on prior LTC use and prior HCE (Table 2). Therefore, it seems imperative to include the latter subgroups in the risk adjustment model to reduce incentives for risk selection.

#### Prior LTC model

Including variables on prior LTC use as risk adjusters by definition reduces the predicted losses on these subgroups to zero. But risk adjustment based on prior LTC use not only reduces predicted losses for prior LTC users but also for many subgroups based on prior HCE and for several subgroups of individuals who were hospitalized for diagnoses that were associated with the highest predicted loss without risk-adjustment (Table 2). This finding implies that it is no longer attractive for insurers to select against any of these groups of patients. For some other subgroups based on prior health care use and on HCE, however, including variables on prior LTC use as risk adjusters does not substantially reduce the predicted losses. Therefore, insurers have an incentive to detect and avoid these subgroups, which are not included in the risk adjustment formula and which are expected to generate a loss to the insurer.

#### Prior HCE and DCG model

Subsequently, we examine the effect of adding information on prior health care use and HCE patterns in the risk adjustment formula on the predicted losses. The predicted losses for the subgroups of insured that used LTC in 2004 all remain above the threshold of 1,000 euro when DCGs are added to the model, along with variables indicating high expenditures (top 15 %) on hospital and outpatient care for the last three and the last five years, and high expenditures on prescription drugs, transport, and durable medical equipment for the last year, the last 3 and the last 5 years (Table 2). But while these variables only have a small impact on the predicted loss for subgroups of LTC users, including HCE is important for reducing the predicted loss for subgroups based on prior hospital admissions for several diagnoses, e.g. heart failure, and asthma and COPD. So while for some diagnoses prior LTC use is more important in reducing the predicted loss, for other diagnoses prior HCE and DCGs causes the largest drop in the predicted losses.

#### Full model

When all information is combined in the full risk adjustment model, the predicted losses are substantially reduced for many of the subgroups we distinguished. For example, this full model reduces predicted losses sufficiently for all but seventeen diagnoses and for all but one type of treatment. Yet, including information on prior HCE and the variables on LTC use also leads to predicted profits larger than 1,000 euro for three diagnoses: hip fracture, chronic ulcers of skin including decubitus (Table 2) and other lower extremity fracture (not in Table 2).

The initial predicted losses also vanish for the subgroups based on self-reported disability, health and socio-economic status when prior LTC use and prior HCE are included in the risk adjustment formula. Although the loss is still larger than 1,000 euro for persons who are unable able to perform at least one ADL, it is no longer significantly different from zero (Table 2).

#### Incentive compatible model

All subgroups based on prior LTC use and listed in Table 2 are large and generate a large predicted loss in the absence of risk adjustment. Yet, some of these subgroups are expected to give insurers perverse incentives because inclusion of enrollees in these subgroups is financially attractive and can be easily manipulated. For example, the required additional spending for admitting a person for a single day in a nursing home (about 190 euro—see Table 3 in Appendix) is much lower than the subsequent increase in the risk-adjusted capitation payment of 11,299 euro for the subgroup of people who are admitted to a nursing home for 1–90 days. Table 3 in Appendix shows that when an individual uses LTC during a given year, in the next year the insurer would be compensated for most of the loss if risk adjustment were based on prior LTC utilization.

The trade-off between incentives for efficiency and incentives for risk selection is also relevant for some subgroups based on prior HCE and health care use. For some subgroups, the inclusion criteria are set at low levels because very few individuals use these services, e.g. individuals with high expenditures on transportation or medical equipment. As a result, for these groups the minimum amount of expenditures is lower than the increase in the risk adjustment payment. Therefore, the subgroups based on only high expenditures in the previous year are omitted in the incentive compatible model. For DCGs and subgroups with high HCE in successive years the incentive problem is expected to be limited (Van de Ven and Ellis 2000).

Leaving subgroups that were expected to compromise insurers’ incentives for efficiency out of the incentive compatible model has a small effect on the overall predictive power of the model: the incentive compatible model has an $\hbox {R}^{2}$-statistic of 0.70, compared to 0.73 for the full model. A comparison of the results of the full model and the incentive compatible model at the subgroups level reveals that removing these risk adjusters does not only affect the predicted losses for the subgroups that are no longer included but also the predicted losses for subgroups based on hospital diagnoses and treatments and for the subgroups based on detailed survey information on health and disability. Yet, the impact on the predicted losses for these other subgroups is often fairly limited. Therefore, further reduction of the number of subgroups in the risk adjustment model may be considered.

## Conclusion and discussion

In the Netherlands and several other countries, public LTC insurance is offered by non-competing agents that are not at risk for providing coverage. This situation is suboptimal because it provides these agents with little or no incentive for efficiency and cost containment. In the Netherlands, the government proposed to incentivize insurers to increase efficiency and innovation of LTC provision by putting them at risk for providing LTC coverage and allowing them to compete for customers and thus let them reap the benefits of improvements in quality and reduced expenditures. Introducing financial risk would be easy but might lead to socially undesired outcomes in terms of equity and efficiency.

To maintain universal access in a competitive LTC insurance market, an adequate system of risk-adjusted premium subsidies is imperative. Without adequate risk adjustment insurers face strong incentives to deter subgroups that generate predictable losses, e.g. by excluding relevant benefits from the benefit package or by lowering the service level or the quality of the contracted provider network that they offer to these subgroups (Cao and McGuire 2003). We have investigated the scope for risk selection and the feasibility of a LTC risk adjustment formula that sufficiently reduces insurers’ financial incentives for risk selection^14^. The attractiveness of managed competition vis-à-vis alternative ways to organize LTC insurance depends inter alia on the ability to prevent risk selection^15^. Little is known, however about the feasibility of adequate risk adjustment for LTC. Hence, improved knowledge about the extent to which risk adjustment can successfully reduce insurers’ incentives for risk selection helps us to better evaluate the feasibility of managed competition in LTC insurance^16^.

Our findings demonstrate that a model that is only based on demographic characteristics performs poorly: subgroups that may be identified based on their prior LTC use, prior HCE or other individual characteristics are predicted to generate large losses to the insurer in case of annual contracts with community rated premiums. This means that in this case, insurers will face very strong financial incentives to discourage these subgroups from joining their plan.

Subsequently, we investigated the impact of (1) including individual-level information on prior health care and LTC use and (2) excluding risk adjusters that compromise insurers’ incentives for efficiency. Not surprisingly, prior use of LTC services is the best available predictor of future LTC use and its inclusion substantially reduces incentives for risk selection. The main drawback of this risk adjuster is that it simultaneously reduces incentives for efficiency. This problem may at least partially be overcome by (i) including indicators for having used LTC for multiple years because it may be harder for insurers to manipulate use and expenditures for multiple subsequent years than for just one year^17^ and (ii) by optimizing the DCGs for predicting LTC expenditures^18^.

An important finding is that in addition to prior LTC use, prior HCE and inpatient diagnosis and treatment information also prove to be vital: predicted losses persist for certain categories of HCE and for some inpatient diagnoses that occur mostly among the frail elderly even when prior LTC use is taken into account. These diagnoses probably indicate a negative health shock that leads to increased formal LTC use. However, including all available risk adjusters in the model does not fully eliminate the potential for risk selection. While the predicted losses disappear for health, disability and socio-economic characteristics that can be obtained from a survey, risk selection on the basis of some inpatient diagnoses and treatments as well as prior LTC use remains feasible. An insurer can easily identify most of these subgroups, e.g. the subgroup of patients who received short-term institutional LTC, were admitted to a hospital for a hip fracture, dementia-related problems or asthma or COPD, or who had high HCE in 2004 but not in 2003 or 2002. Yet, including these variables in the risk adjustment formula is not an option, as it would give insurers an incentive to overprovide these types of health care.

Ideally, risk adjustment is based on data on individuals’ underlying needs for care but such information is rarely included in administrative data and insurers’ LTC claims data. As a consequence, in the Netherlands and elsewhere, risk adjustment in LTC will have to rely on prior utilization and expenditure data, which is likely to not only reduce incentives for risk selection but also incentives for efficiency. Most of all, our findings highlight the interrelatedness of elderly care, medical care and social care. This implies that, in order to prevent risk selection, any risk adjustment formula needs to take into account the potential simultaneous or subsequent use of these other types of care. Therefore, our findings also have implications for the reverse relationship: taking into account prior LTC use should also be considered and studied for optimizing risk adjustment in health insurance.

## Appendix

See appendix Tables 3,  4,  5, 6 and  7.

**Table 3:** Coefficients of risk classes included in the risk-adjustment model

	Prevalence	Demographic model	Prior LTC model	Prior HCE and DCG model	Full model	Incentive compatible model	Min. cost of prior use to qualify for subgroup
Demographic information		Yes	Yes	Yes	Yes	Yes	
Prior LTC use		No	Yes	No	Yes	Yes	
Prior HCE and diagnosis information		No	No	Yes	Yes	Yes	
Female age $<$ 50	26.43	$-$346*	$-$49*	$-$307*	$-$46*	$-$73*	
Female age: 50–64	9.54	327*	30*	147*	$-$7	22*	
Female age: 65–69	2.70	929*	169*	576*	101*	154*	
Female age: 70–74	2.47	2,125*	404*	1,569*	317*	460*	
Female age: 75–79	2.26	4,733*	928*	3,937*	832*	1,135*	
Female age: 80–84	1.89	9,469*	1,863*	8,444*	1,770*	2,286*	
Female age: 85–89	1.13	17,034*	3,310*	15,770*	3,220*	3,946*	
Female age: 90+	0.68	27,154*	4,556*	25,732*	4,481*	5,403*	
Male age $<$ 50	31.01	0	0	0	0	0	
Male age: 50–64	12.44	188*	47*	55*	17*	22*	
Male age: 65–69	2.96	592*	210*	313*	154*	148*	
Male age: 70–74	2.51	1,256*	401*	853*	329*	370*	
Male age: 75–79	1.90	2,698*	793*	2,106*	700*	847*	
Male age: 80–84	1.25	5,473*	1,460*	4,671*	1,350*	1,678*	
Male age: 85–89	0.58	9,961*	2,333*	8,999*	2,229*	2,784*	
Male age: 90+	0.25	16,478*	3,005*	15,384*	2,924*	3,631*	
Male living alone	10.63	239*	$-$361*	213*	$-$358*	$-$158*	
Female living alone	11.30	2,096*	72*	1,917*	68*	339*	
$\ge$1 h per week in 2004: domestic care I	0.81		336*		318*	1,534*	2,360.80
$\ge$1 h per week in 2004: domestic care II	1.87		2,594*		2,548*	3,865*	733.20
$\ge$1 h per week in 2004: activating support	0.06		885*		857*	1,082*	1,367.60
$\ge$1 h per week in 2004: guidance	0.16		4,760*		4,783*	5,369*	2,298.40
$\ge$1 h per week in 2004: personal care	1.44		7,346*		7,275*	9,101*	2,033.20
$\ge$1 h per week in 2004: nursing	0.43		12,079*		11,840*	13,502*	3,088.80
Residential home 1–90 days	0.18		6,192*		6,112*		86.02
Residential home 91–180 days	0.07		11,869*		11,715*	15,147*	7,827.82
Residential home 181–365 days	0.13		20,595**		20,458*	27,012*	15,569.62
Residential home 366 days	0.78		22,901*		22,728*	28,010*	31,483.32
Nursing home: somatic care 1–90 days	0.02		11,578*		11,299*		188.65
Nursing home: somatic care 91–180 days	0.01		24,051*		23,572*	26,345*	17,166.87
Nursing home: somatic care 180–365 days	0.01		41,468*		41,025*	47,521*	34,145.09
Nursing home: somatic care 366 days	0.02		58,101*		58,089*	63,727*	69,044.77
Nursing home: psychogeriatric care 1–90 days	0.01		29,897*		29,773*		203.70
Nursing home: psychogeriatric care 91–180 days	0.01		37,814*		37,798*	41,849*	18,535.95
Nursing home: psychogeriatric care 180–365 days	0.01		54,145*		54,055*	59,739*	36,868.21
Nursing home: psychogeriatric care 366 days	0.04		63,126*		63,206*	68,788*	74,551.19
Nursing home: combined care 1–90 days	0.18		20,167*		19,956*		247.01
Nursing home: combined care 91–180 days	0.07		37,289*		36,939*	38,841*	22,477.55
Nursing home: combined care 180–365 days	0.08		59,337*		59,072*	63,692*	44,708.10
Nursing home: combined care 366 days	0.22		78,034*		78,092*	83,659*	90,404.23
Used home care on the last day of the year	3.01		2,750*		2,609*		14.10
Used institutional care on the last day of the year	2.09		6,366*		6,279*		86.02
In top 15 % in prior year: durable medical equipment	12.12			156*	79*		1
In top 15 % in prior 3 years: durable medical equipment	5.78			1,471*	353*		3
In top 15 % in prior 5 years: durable medical equipment	3.69			1,980*	370*	469*	5
In top 15 % in prior year: transportation	3.52			3,780*	579*		1
In top 15 % in prior 3 years: transportation	0.76			5,802*	209*		3
In top 15 % in prior 5 years: transportation	0.39			4,649*	190*	670*	5
In top 15 % in prior year: paramedical care	4.30			532*	$-$51*		1
In top 15 % in prior 3 years: paramedical care	1.60			1,064*	172*		212.77
In top 15 % in prior 5 years: paramedical care	0.90			1,425*	371*	471*	382.81
In top 15 % in prior year: drugs	12.26			$-$203*	21*		588.88
In top 15 % in prior 3 years: drugs	8.43			$-$84*	55*		1,761.78
In top 15 % in prior 5 years: drugs	6.66			$-$162*	33*	64*	2,743.73
In top 15 % in prior 3 years: hospital + outpatient care	2.41			69*	$-$4	96*	2,096.22
In top 15 % in prior 5 years: hospital + outpatient care	1.01			109*	88*	136*	3,145.02
No records available for all of the last 5 years	25.37			323*	76*	62*	0
No DCG	97.60			0	0	0	
DCG 1	0.39			$-$883*	$-$1,518*	$-$116*	
DCG 2	0.49			$-$861*	$-$81*	243*	
DCG 3	0.25			$-$1,070*	$-$83*	287*	
DCG 4	0.20			5,323*	986*	3,373*	
DCG 5	0.31			882*	312*	953*	
DCG 6	0.19			331*	148*	689*	
DCG 7	0.13			2,295*	1,068*	1,730*	
DCG 8	0.12			1,972*	809*	1,445*	
DCG 9	0.13			1,772*	805*	1,397*	
DCG 10	0.09			4,313*	3,093*	3,995*	
DCG 11	0.05			4,770*	3,647*	4,540*	
DCG 12	0.05			2,154*	1,635*	2,327*	
Intercept		$-$3	6*	$-$207*	$-$36*	$-$18*	
$\hbox {R}^{2}$		0.23	0.73	0.24	0.73	0.70	
Number of observations		5,719,934	5,719,934	5,719,934	5,719,934	5,719,934	

* Significantly different from zero at the *p* $<$ 0.05 level

**Table 4:** Predicted losses for subgroups of LTC users in prior year

	No risk adjustment	Demographic model	Prior LTC model$^{\mathrm{a}}$	Prior HCE and DCG model	Full model$^{\mathrm{a}}$	Incentive compatible model$^{\mathrm{a}}$	Subgroup size
Demographic information	No	Yes	Yes	Yes	Yes	Yes	
Prior LTC use	No	No	Yes	No	Yes	Yes	
Prior HCE and diagnosis information	No	No	No	Yes	Yes	Yes	
Activating support	3,763	3,556		3,080			3,559
Domestic care I	5,006	$-$3,232		$-$3,524			46,611
Domestic care II	9,753	2,147		1,479			10,7181
Guidance	21,909	11,406		10,617			9,252
Personal care	16,992	8,293		7,269			82,116
Nursing	25,799	17,183		15,303			24,727
Residential home 1–90 days	22,224	11,978		10,488		8,207	10,054
Residential home 91–180 days	34,768	22,153		20,826			3,903
Residential home 181–365 days	36,137	23,203		22,153			7,655
Residential home 366 days	31,190	14,777		14,139			44,726
Nursing home: somatic care 1–90 days	24,292	16,859		13,829		13,104	1,042
Nursing home: somatic care 91–180 days	38,721	30,839		27,338			389
Nursing home: somatic care 181–365 days	54,610	46,047		42,608			376
Nursing home: somatic care 366 days	65,995	56,700		55,923			1,111
Nursing home: psychogeriatric care 1–90 days	51,386	41,756		40,013		32,614	755
Nursing home: psychogeriatric care 91–180 days	62,188	52,102		51,072			385
Nursing home: psychogeriatric care 180–365 days	68,104	57,465		56,406			517
Nursing home: psychogeriatric care 366 days	70,693	58,546		58,887			2,049
Nursing home: combined care 1–90 days	31,009	23,310		20,888		21,005	10,546
Nursing home: combined care 91–180 days	51,350	42,482		39,803			3,853
Nursing home: combined care 180–365 days	71,408	61,349		59,290			4,359
Nursing home: combined care 366 days	85,868	74,655		74,693			12,439
Receiving home care on last day of 2004	10,078	2,459		1,669		876	17,2291
Stay in LTC institution on last day of 2004	31,213	21,576		20,881		3,155	119,785

All reported predicted losses are significant at the *p* $<$ 0.05 level

$^{\mathrm{a}}$ Cells are empty if this variable is included in this risk adjustment model

**Table 5:** Predicted losses for subgroups based on HCE in 2000–2004

	No risk adjustment	Demographic model	Prior LTC model	Prior HCE and DCG model$^{\mathrm{a}}$	Full model	Incentive compatible model$^{\mathrm{a}}$	Subgroup size
Demographic information	No	Yes	Yes	Yes	Yes	Yes	
Prior LTC use	No	No	Yes	No	Yes	Yes	
Prior HCE and diagnosis information	No	No	No	Yes	Yes	Yes	
In top 15 % in prior 3 years: hospital + outpatient care	3,410	1,804	278				137,618
In top 15 % in prior 5 years: hospital + outpatient care	4,031	2,321	393				57,597
Expenditures on medical equipment in prior year	4,108	949	185			93	693,144
Expenditures on medical equipment in prior 3 years	6,524	1,846	320				330,813
Expenditures on medical equipment in prior 5 years	7,259	2,057	326				211,030
Expenditures on transportation in prior year	7,350	4,271	582			916	201,609
Expenditures on transportation in prior 3 years	9,040	5,753	353				43,615
Expenditures on transportation in prior 5 years	8,565	5,347	374				22,462
Expenditures on paramedical care in prior year	2,409	1,319	107			53	246,233
In top 15 % in prior 3 years: paramedical care	3,635	2,009	350				91,743
In top 15 % in prior 5 years: paramedical care	4,371	2,338	462				51,218
In top 15 % in prior year: pharmaceuticals	2,832	572	130			28	701,221
In top 15 % in prior 3 years: pharmaceuticals	3,215	678	140				482,222
In top 15 % in prior 5 years: pharmaceuticals	3,448	742	147				381,160
No prior HCE available	$-$445	25	17				1,450,876
DCG 1	2,080	$-$272	$-$1,474				22,198
DCG 2	1,921	466	117				27,820
DCG 3	2,400	349	117				14,018
DCG 4	10,172	6,865	1,145				11,358
DCG 5	5,109	2,189	471				17,981
DCG 6	3,821	1,346	276				10,586
DCG 7	4,595	3,512	1,215				7,391
DCG 8	6,187	3,881	1,033				7,027
DCG 9	4,787	3,231	9,78				7,459
DCG 10	7,602	5,840	3,275				5,365
DCG 11	7,461	6,220	3,823				2,939
DCG 12	5,275	3,536	1,805				2,684

All reported predicted losses are significant at the *p* $<$ 0.05 level

$^{\mathrm{a}}$ Cells are empty if the variable is included in this risk adjustment model

**Table 6:** Predicted losses for subgroups based on diagnosis information from 2004 hospital admission data

Diagnosis in 2004	No risk adjustment	Demographic model	Prior LTC model	Prior HCE and DCG model	Full model	Incentive compatible model	Subgroup size
Demographic information	No	Yes	Yes	Yes	Yes	Yes	
Prior LTC use	No	No	Yes	No	Yes	Yes	
Prior HCE and diagnosis information	No	No	No	Yes	Yes	Yes	
Ten diagnoses with the highest initial predicted loss							
Dementia	30,423*	20,777*	**9,255***	20,114*	9,274*	11,692*	821
Hip fracture	21,225*	12,029*	$-$ **950***	9,167*	$-$1,229*	3,205*	6,433
Parkinson’s disease	21,093*	17,566*	**6,486***	13,817*	5,730*	7,650*	510
Chronic ulcers of skin including decubitus	13,421*	8,676*	$-$ **224**	4,514*	$-$1,897*	$-$1,528*	873
Stroke	10,840*	7,288*	**1,109***	2,219*	327*	1,271*	11,998
Septicaemia	10,620*	7,322*	**1,100***	4,277*	592	2,330*	679
Heart failure	10,054*	3,806*	858*	**745***	182	637*	8,147
Pancreas cancer	9,584*	7,556*	6,316*	**2,605***	3,525*	3,469*	329
Osteoporosis	9,334*	4,590*	**1,002***	2,710*	662*	1,660*	1,598
Acute renal and urinary infections	8,908*	5,452*	**1,252***	3,420*	954*	1,798*	3,942
Ten diagnosis with the highest initial predicted loss for which the incentive compatible model sufficiently reduced the predicted loss							
Heart failure	10,054*	3,806*	858*	**745***	182	637*	8,147
Diseases of the blood and bloodforming organs	8,139*	3,713*	**1,191***	1,718*	494*	755*	6,814
Diabetes mellitus including diabetic complications	6,478*	4,633*	**679***	2,770*	133	231	4,746
Stomach cancer	6,148*	3,900*	2,673*	$-$ **290**	368	553	664
Asthma and COPD	6,128*	3,721*	876*	**122**	$-$78	26	8,035
Nephritis and nephropathy	5,579*	3,906*	**535***	767*	$-$189	128	2,117
Esophagus cancer	5,429*	3,768*	2,184*	$-$ **1,207**	$-$725	$-$343	426
Colorectal cancer	5,416*	2,438*	1,095*	$-$ **20***	235	464	3,975
Other endocrine, nutritional and metabolic diseases	4,504*	2,829*	**761***	1,440*	474*	849*	8,510
Congenital anomalies of nervous system	4,082	2,558	**181**	546	$-$239	$-$229	50

* Significant at the *p* $< 0.05$ level; *bold numbers* indicate whether including prior LTC use or prior HCE and diagnosis information decreased the predicted loss most. Results for subgroups based on primary treatment information from 2004 and from earlier years and for subgroups based on primary diagnoses from earlier years available upon request

**Table 7:** Predicted losses for subgroups based on the General Survey of Living Conditions (POLS) 2004

	No risk adjustment	Demographic model	Prior LTC model	Prior HCE and DCG model	Full model	Incentive compatible model	Subgroup size	Prevalence$^{\mathrm{a}}$
Demographic information		Yes	Yes	Yes	Yes	Yes		
Prior LTC use		No	Yes	No	Yes	Yes		
Prior HCE and diagnosis information		No	No	Yes	Yes	Yes		
From POLS survey (n $=$ 7,790)								
Self-rated health: bad	4,591*	3,167*	421	2,443*	294	613	482	6.19
Long-term illness	1,185*	380	32	186	$-$5	49	2,927	37.60
Education: low	1,314*	193	9	166	8	72	2,616	33.95
Education: high	$-$834*	$-$245*	$-$20	$-$240*	$-$23	-86	1,006	13.05
GALI problems$^{\mathrm{b}}$	2,912*	1,405*	134	986*	67	200	1,327	17.05
Income: lowest quartile	661*	488*	95	474*	91	119	1,936	
Income: highest quartile	$-$743*	$-$33	$-$1	6	3	$-$12	1,945	
From POLS survey (n = 3,619)								
Specialist visit in 2004	679*	1,57	11	$-$7	$-$19	$-$76	1,584	43.77
Use of pharmaceuticals in prior 14 days	1,039*	265	$-$158	25	$-$207	$-$294*	476	13.16
Physiotherapy	1,042*	738*	$-$9	509	$-$36	$-$40	738	20.39
At least 1 chronic disease	270	111	63	101	60	10	994	34.33
At least 2 chronic diseases	362*	223	86	218	85	46	456	12.59
Blindness	638	$-$318	$-$189	$-$442	$-$209	$-$258	536	17.96
Deafness	1,370*	$-$148	$-$124	$-$176	$-$134	$-$144	466	15.66
Diabetes	1,509	$-$670	$-$60	$-$1,153	$-$129	$-$320	121	4.05
Cancer	2,621*	968	$-$26	588	$-$122	$-$319	181	6.07
Stroke	4,349*	1,404	$-$64	1,161	$-$165	$-$302	108	3.61
Blood pressure	1,801*	204	34	133	0	$-$53	405	13.50
Ciculatory system	2,848*	466	$-$145	$-$146	$-$246	$-$477	77	2.57
Urinary incontinence	3,131*	1,283	683	911	631	688	166	5.53
Osteoarthritis	1,649*	$-$126	$-$78	$-$266	$-$105	$-$124	445	14.82
Arthritis	2,025*	451	$-$61	315	$-$98	$-$75	191	6.37
At least 1 ailment	198	$-$107	$-$66	$-$152	$-$77	$-$128*	1,659	21.30
At least 2 ailments	752*	$-$49	$-$133	$-$167	$-$157	$-$194	917	11.77
PCS-12$^{\mathrm{c}}$ score $<$30	3,746*	1,834	$-$308	1,326	$-$397	$-$219	208	7.62
Has difficulty to/cannot perform $\ge$1 ADL$^{\mathrm{d}}$	16,221*	8,918*	357	7,887*	289	532	90	7.45
Cannot perform $\ge$1 ADL	23,365*	13,476*	1,479	11,906*	1,409	1,365	32	2.65
Has difficulty to/cannot perform $\ge$1 mobility task	9346*	3,627*	212	2,953*	156	37	195	16.28
Cannot perform $\ge$1 mobility task	15,394*	7247*	464	6,225*	388	656	69	5.76

$^{\mathrm{a}}$ Prevalence among respondents for whom this characteristic is not missing

$^{\mathrm{b}}$ Global Activity Limitation Indicator (Van Oyen et al. 2006)

$^{\mathrm{c}}$ Physical Component Scale (Ware et al. 1996)

$^{\mathrm{d}}$ Activity of daily living: dressing; walking across a room; bathing; eating; getting in / out of bed; using the toilet (Katz and Akpom 1976)

*Predicted loss is not significant at the *p* $<$ 0.05 level

## Footnotes

1 Definitions of LTC vary internationally. In this paper we focus on elderly care, which in the Netherlands (and elsewhere) accounts for the majority of total LTC expenditure covered by LTC insurance (CVZ 2011). Elderly care is defined as home care, social assistance, assistance with activities of daily living and inpatient stays in either a residential home or a nursing home. This definition comprises both “medical” and “non-medical” LTC: unlike in some other European countries, in the Netherlands there is no sharp distinction between medical and non-medical LTC.

2 See Van de Ven and Schut (2011) for a description of how these subsidies are organized and for a full overview of strategies that are used to ensure affordable access to coverage.

3 Insurers may engage in risk selection by differentiating their benefit packages or, if the benefit package is fixed, the level of service or the quality of the contracted provider network that they offer to each type of patient (Cao and McGuire 2003). Thus, an insurer may discourage individuals who need or desire a particular service to join its plan by limiting access or by contracting unattractive providers. Risk selection is undesirable because it may lead to welfare losses if (i) resources are employed for risk selection rather than for improving care; (ii) inefficient health plans that are successful in risk selection survive; and/or (iii) good quality LTC is underprovided (Van de Ven and Ellis 2000).

4 See De Meijer et al. (2011) for a more detailed description of the data.

5 Until 2006 enrollment was mandatory for two thirds of the population with an income below a threshold; the remainder of the population was not eligible for social health insurance and could buy private insurance. By contrast, public LTC insurance was (and is) mandatory for the entire population.

6 The selection of subgroups is based on data availability.

7 Other commonly used specifications did not provide a strictly better fit than OLS. Results for the other specifications are available from the corresponding author.

8 The remaining 5 % consisted of cash transfers, which are not in the dataset.

9 These four categories include domestic care I—cleaning, domestic care II—cleaning and help with organizing the household, personal care and nursing. Information on assistance and support is not available for 2005.

10 As for LTC, risk adjustment based on health care use rather than expenditures would reward insurers for negotiating lower prices but data on health care use was not available, except for the data needed to construct Diagnostic Cost Groups.

11 The assigned DCG does not match with the actual DCG for some individuals because of two limitations of the data set: 1. not all hospitals reported information on patients to the national medical registry; 2.information on two relevant ‘side treatments’, dialysis and artificial respiration at home was not available at all. As a consequence, DCG 13 (dialysis) is empty and the reference category consists of DCG0, DCG13 and patients who needed artificial respiration and should therefore be in DCG12. Furthermore, information on radiotherapy and chemotherapy was not specific enough to ensure that no patients who do not belong in the related DCG are excluded.

12 Table 3 in the appendix contains descriptive statistics and all regression results.

13 Tables 4–7 in the Appendix contain the predicted losses for all the subgroups that were included in the final model, 20 subgroups based on diagnosis from information on hospital admissions in 2004 and subgroups that were based on the POLS survey data; results for other subgroups are available from the corresponding author.

14 An alternative solution to the problems caused by risk selection would be to deny LTC users to switch from one insurer to another, yet this alternative would substantially reduce the insurer’s incentives to act as prudent buyers of LTC for this group and would increase their incentives to keep out future LTC users if risk adjustment is inadequate.

15 In addition to the incentives for efficiency and for meeting consumer preferences that managed competition may create for insurers, it may also affect overall efficiency by facilitating innovation through trial-and-error by insurers.

16 Risk adjustment may not only be used to equalize insurer payments and their expected costs but also in the context of capitated and bundled provider payments.

17 Risk adjustment based on multiple years of use may be more useful for home care than for institutional care because of the limited average length of stay at a care facility.

18 In addition, incentives for risk selection may be reduced by including more subgroups, e.g. based on socio-economic status and more specific information on prior use of durable medical equipment that indicates disability (see e.g. Van Kleef and Van Vliet 2010), as risk adjusters.
